# The encoding of stochastic regularities is facilitated by action-effect predictions

**DOI:** 10.1038/s41598-021-86095-4

**Published:** 2021-03-24

**Authors:** Betina Korka, Erich Schröger, Andreas Widmann

**Affiliations:** 1 Cognitive and Biological Psychology, Institute of Psychology – Wilhelm Wundt, Leipzig University, Neumarkt 9-19, 04109 Leipzig, Germany; 2 Leibniz Institute for Neurobiology, Magdeburg, Germany

**Keywords:** Auditory system, Cognitive neuroscience, Perception

## Abstract

Our brains continuously build and update predictive models of the world, sources of prediction being drawn for example from sensory regularities and/or our own actions. Yet, recent results in the auditory system indicate that stochastic regularities may not be easily encoded when a rare medium pitch deviant is presented between frequent high and low pitch standard sounds in random order, as reflected in the lack of sensory prediction error event-related potentials [i.e., mismatch negativity (MMN)]. We wanted to test the implication of the predictive coding theory that predictions based on higher-order generative models—here, based on action intention, are fed top-down in the hierarchy to sensory levels. Participants produced random sequences of high and low pitch sounds by button presses in two conditions: In a “specific” condition, one button produced high and the other low pitch sounds; in an “unspecific” condition, both buttons randomly produced high or low-pitch sounds. Rare medium pitch deviants elicited larger MMN and N2 responses in the “specific” compared to the “unspecific” condition, despite equal sound probabilities. These results thus demonstrate that action-effect predictions can boost stochastic regularity-based predictions and engage higher-order deviance detection processes, extending previous notions on the role of action predictions at sensory levels.

## Introduction

Information from various sources constantly arrives at our senses, some of the information is caused by our own actions, and other is of different origin. In order to make sense of it, our brains need to continuously build relationships between sensory events and between our actions and sensory events. Understanding the precise mechanisms that allow us to form complex models of the world has gathered considerable support for predictive coding, a unifying brain theory based on the free energy principle which largely states that our brains are optimized to predict future events^1–5^. Brain predictions are derived at multiple levels, for instance from detected sensory regularities or based on learned action-effects. In this paper, we focus on auditory predictions, as the auditory system has been particularly investigated in relationship to its ability to detect simple and complex regularities^6^, but also in relationship to action intention and learned action-effects^7,8^. Here, we aim to further understand the relationship between sensory-regularity-based and action-effect-regularity-based predictions (or in short, sensory and action predictions), particularly, whether action-effect expectations can help detect sensory regularities that are otherwise difficult to be detected. In other words, can one type of auditory prediction facilitate/compensate for the other?

In oddball paradigms, regularity detection is usually investigated by looking at stimuli that deviate from an established rule, which elicit the well-known *mismatch negativity* (MMN), a negative deflection typically observed at latencies between 100 and 250 ms post stimulus presentation by subtracting the standard-evoked from the deviant-evoked activity. Probably one of the most investigated ERP components since its discovery^9^, the MMN has increasingly been interpreted from a predictive coding perspective^3,10–13^. In short, the predictive coding theory postulates that higher cortical areas send prediction signals regarding the expected input to the lower areas, which, in turn, send prediction error signals back up the cortical hierarchy, in case the expected and the received input do not match. In this context, the MMN arises as the result of a comparison between the top-down expectations and bottom-up sensory input. Thus, it represents a marker of prediction error following mismatching stimuli.

The MMN is typically investigated in relationship to regularity violations, and outside the context of action. Yet in one study^14^, participants were asked to randomly press left and right keys to generate tones A and B, which were inversely associated with the two key-presses (i.e., the left key-press frequently generated tone A, while right key-press frequently generated tone B). Rarely, these learned associations were violated by presenting the other tone i.e,. the tone associated with the other key-press. Important to note here is that the two tones were overall presented with equal chances i.e., there was no overall sensory regularity (only an action-effect regularity). In a second condition, participants generated a typical oddball sequence where the left and right keys triggered tone A frequently and tone B rarely, regardless of the key-press choice (no action-effect regularity, only a sensory regularity). Interestingly, similar MMNs were found in both conditions, suggesting that the predictive system behind the MMN generation is able to rely on both sensory regularity-based predictions, as well action predictions based on the intention to generate specific action-effects^14^.

Nevertheless, one interesting limitation of the MMN system has been recently brought forward: it seems to struggle with recognizing stochastic regularities^15^. That is, when two highly probable standards (presented randomly, each in 45% probability) enclosed the pitch of a rare deviant (presented randomly, with 10% probability), the MMN was consistently failed to be observed. This was not the case when the deviant’s pitch was excluded from the distribution defined by the two standards, or if the deviant was presented within a deterministic sequence of alternating standards. The results were interpreted from a predictive coding perspective and in agreement with previous findings^16,17^ suggesting that predictions are based on a distribution of possible outcomes. However, this means that sounds from within the distribution body, no matter how rare, will not be identified as prediction violating. The authors of this study thus suggested that it seems like the brain did not quickly learn that two outcomes are more likely than a third one, as a gambler would easily do^15^.

While this appears to be a surprising limitation, it might be that the detection of more complex (stochastic) regularities requires a stronger generative model and/or predictions at higher levels. After all, gambling involves acting with the intention to achieve expected outcomes, rather than just passive recognition of probabilities. In line with this, we were interested to see whether associating the two likely outcomes (i.e., the standard tones) with intended action-effects would lead to detection of the stochastic regularity violation. This was compared to a condition in which both key-presses generated both standard tones, equally likely. We expected that the rare enclosed deviant would lead to mismatch response(s) only when violating the expected action-effects, despite the two conditions being in fact identical in terms of physical stimulation, i.e. the overall as well as transitional probabilities of the three tones did not differ. This would first, extend previous findings indicating that intention leads to predictions at sensory levels^14^ and second, confirm our hypothesis that action-effect expectations can help detect sensory regularities that are otherwise difficult to be detected.

Action predictions are often described in terms of filtering out the expected events via a feedforward mechanism that sends information from the motor to the sensory systems^7,8,18^. Yet, it has recently been argued that arbitrary and rapidly learned action predictions (such as is the case with key-tone associations), by contrast to more body-related predictions (e.g., experiencing pressure), should be best explained by inferential, cognitive processes rather than by motor-based mechanisms^19^. From this perspective, the action and sensory prediction mechanisms become more comparable; indeed, functional equivalence between the two has previously been proposed^20,21^. Given these alternative views on the correspondence between action and sensory predictions, this study should additionally offer important insights regarding the role of action predictions relative to that of (complex) sensory predictions.

Finally, while most action prediction studies look at the effects of sound predictability at post-stimulus intervals, an interesting question is whether the specificity of the action-sound associations differently modulates the pre-stimulus, action preparation stages. While such a finding would provide important new directions, research on the topic remains scarce—in fact, to the best of our knowledge, the direct relationship between action preparation and action-effect specificity remains uncharted. We thus additionally address this in a secondary, exploratory analysis by focusing on the lateralized readiness potential (LRP), a component presumably reflecting the preparation of a specific hand (or foot) action^22^.

## Materials and method

### Participants

Data were collected from 14 healthy participants (8 male, mean age: 23.3 year-old, age range: 19–30 year-old) who gave a written informed consent for the study participation; all participants reported normal hearing, normal-to-corrected vision, no history of neurological conditions, nor regular intake of any prescribed drugs. All participants were right-handed. The Ethics Advisory Board of Leipzig University approved the study procedure, in agreement with the Declaration of Helsinki (code of approval: 2020.02.24_eb_41). Participants received either compensation of 8 euros/hour or course credits.

### Apparatus and stimuli

For the whole experiment duration, participants sat in a comfortable office chair in an electrically-shielded, double-walled sound booth (Industrial Acoustics Company). During each of the experimental blocks, participants were required to place their head into a chin and forehead rest, in order to minimise EEG-related artifacts. As in^15^, stimuli were simple sine wave tones with fundamental frequencies of 1000 Hz for the deviant and 900 Hz and 1100 Hz for the standards, respectively. The tones had a duration of 50 ms including 5-ms rise-and-fall times and were presented binaurally over a pair of headphones (Sennheiser HD 25) at an intensity level of 82 dB SPL. The two keys to be pressed had dimensions of 3.8 × 4.5 cm and were placed on the desk in front of the participant. Importantly, we used custom‐built infrared photoelectric sensor‐based keys which have the advantage of being completely silent while still providing tactile feedback, by comparison to typical membrane or mechanical keys. During each block, participants were required to fix their gaze on a fixation cross which was displayed on a 19-in. CRT monitor (G90fB, ViewSonic, resolution 1024 × 768 pixels, refresh rate of 100 Hz), which was placed at a comfortable seeing distance in front of the participant (∼60 cm). The experiment was implemented in Psychtoolbox 3^23^, in combination with GNU Octave Version 4.0.0, running on Linux OS.

### Task

**Figure 1:**
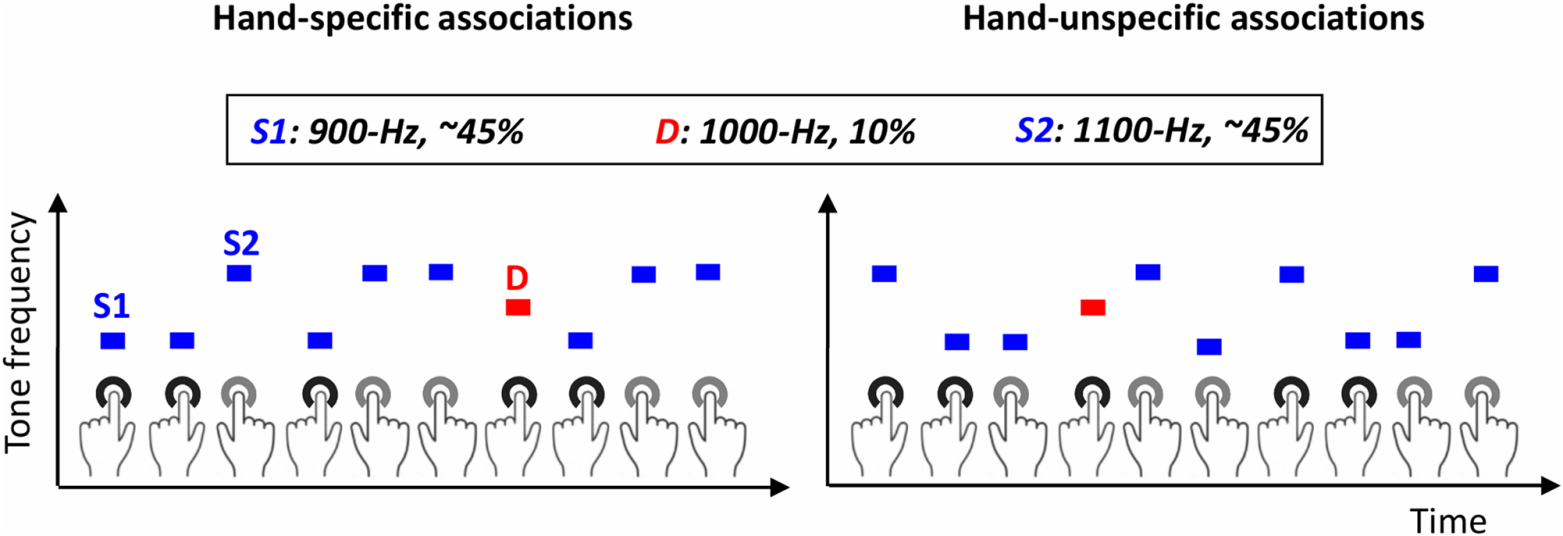
Experimental conditions. In the case of hand-specific associations (left), the left key-press generated the standard lower 900-Hz tone and the right key-press generated the standard higher 1100-Hz tone. In the condition with hand-unspecific associations, both key-presses generated both standard tones with equal chances. In both conditions, the standards were presented with 90% probability (each in about ~ 45% of the trials), while the middle-pitch enclosed 1000-Hz deviant was presented in the remaining 10% of the cases. Participants fixed their gaze on a fixation cross and pressed a key of their choice every second, while avoiding to produce fixed sequences and ensuring that the two keys were pressed equally often throughout one block. The two keys (here, displayed in dark and light grey for the left and right hands for easy discrimination) were completely silent while still providing tactile feedback (i.e., were infrared photoelectric sensor‐based keys, dimensions: 3.8 × 4.5 cm). MS Office PowerPoint 2016 (https://www.office.com) was used to generate this figure and edit subsequent figures.

Figure 1 summarizes the experimental conditions. Participants pressed the left and right keys with their index fingers to generate tones according to the condition-specific instructions. In the condition with *hand-specific associations* (hereafter *SPEC*), participants pressed the left key to produce the lower 900-Hz tone and the right key to produce the higher 1100-Hz tone, which were presented in 90% of the cases (each tone accounting for about 45% of the trials). Rare presentation of the 1000-Hz deviant tone accounted for the remaining 10% of the trials. Here, the *left hand—low tone* and *right hand—high tone* associations regarding the frequently presented tones were kept fixed for all participants, as pre-existent representations regarding size (operationalized here as tone frequency) are likely to be mapped on a left–right continuum. This would thus help create stronger expectations regarding the tone identity following the left and right key-presses. Note that such a relationship between mental spatial representations and both mathematical decisions^24^ as well as pitch-related decisions^25^ has been demonstrated before. In the condition with *hand-unspecific associations* (hereafter *UNSPEC*), both the left and right key-presses produced either the 900-Hz tone or the 1100-Hz tone with equal chances, thus making the key-press choice uninformative with regards to the identity of the forthcoming come. As in the *SPEC* condition, these two tones were presented on 90% of the trials (each with 45% overall probability), while on the remaining 10% of the trials, the rare 1000-Hz deviant was presented instead. The two conditions may also be described in terms of transitional probabilities. That is, while they do not differ in terms of sensory probabilities, the action-sound probabilities are different, this information representing an additional top-down source whose effect is of interest here.

In both conditions, participants’ task was to press the two keys with equal chances throughout one block; regarding the timing, a key was to be pressed every one second while avoiding to produce fixed left/right patterns of key-presses. The time between consecutive key-presses was monitored online. If participants’ pace was much faster or slower than indicated (± 500 ms relative to 1000 ms), a corresponding error message (“Too slow/Too fast”) was presented instead of the tone; this was displayed on the screen underneath the fixation cross after which participants proceeded normally to the next key press. Note that the timing errors did not affect the total number of collected trials for the standard and deviant tones, as error trials were repeated so that the planned number of “correct” timing trials and corresponding tones was insured. In order to have an estimate of task compliance, timing errors were analysed offline as error percentage (%ERR) from the total number of trials (i.e., “correct” timing + error trials), along with the total ratio of left/right key-presses.

Twenty experimental blocks (10 for each condition) were recorded in total. The duration of one block was about 1.5 min, and participants could take self-paced breaks in between. Each experimental block consisted of 90 standard tones and 10 deviant tones. Two shorter practice blocks each consisting of 36 standard tones and 4 deviant tones were performed in the beginning of every condition. The condition order was counterbalanced i.e., half of the participants started with the *SPEC*, and half with the *UNSPEC* condition, while the blocks belonging to the same condition were run one after another. The tone onset immediately followed the key-press (with a delay of ~ 5 ms), the total trial duration being about one second as a function of the participants’ self-pacing. For the whole duration of a block, participants fixed their gaze on the fixation cross presented in the middle of the screen. At the end of every experimental block, feedback regarding the timing (including the percentage of trials with “correct”, “too slow” and “too fast” responses, but also the average timing in ms), as well as the ratio of left-to-right key-presses was displayed on the screen for a duration of 10 s, to help participants maintain/adjust their performance. Only during the practice blocks and starting from the second trial, the timing between consecutive key-presses was additionally displayed on the screen, underneath the fixation cross—this had the purpose of helping participants acquire the desired pace. All information presented on the screen including fixation cross and feedback were displayed in light grey on a dark grey background. The order of standard and deviant tones within a block (and within the same key for the *SPEC* condition) was randomized, with the constraint that the first two tones were always standards and no deviants were presented consecutively.

Finally, in a second part of the experiment, participants performed a passive listening task in which they heard the *stochastic sequences* generated in the active part. This was compared to a *deterministic sequence* of alternating standards, with rare and random deviants in between, as in the original study comparing the two regularity types^15^. For details regarding the passive listening task including method and results, see the Supplementary Information.

### EEG data recording and preprocessing

EEG data were continuously recorded at a sampling rate of 500 Hz with a system equipped with 32 Ag–AgCl active electrodes, using a BrainAmp amplifier and the Vision Recorder software (Brain Products GmbH, Munich, Germany). Two electrodes were placed on the mastoids. One electrode placed on the tip of the nose served as online reference, a ground electrode was placed on the forehead, while three electrodes were used to record EOG activity, two of which were placed on the left and right outer canthi, and one below the left eye. The remaining electrodes were mounted in an elastic cap (actiCAP) following the extended international 10–20 system^26^.

The EEG preprocessing was carried out using the EEGLAB MATLAB-based software^27^. Data were first filtered using a 0.1 Hz high-pass and a 45 Hz low-pass windowed sinc finite impulse response (FIR) filter (Hamming window, filter order 8250—high-pass, and 166—low-pass), in accordance with current recommendations^28^. Channels containing extreme amplitudes were removed using a deviation criterion (threshold = 3) which “calculates the robust *z* score of the robust standard deviation for each channel”^29^; on average, 0.3 channels were excluded (range: 0–2). Data were then epoched around the tone presentation/key-press, separately for the post-stimulus (− 200 to 600 ms) and pre-stimulus (− 850 to 200 ms) analyses. Epochs with amplitudes exceeding a 500 μV amplitude difference threshold were removed; on average, 4.8 epochs were excluded at this step (range: 0–36). An Independent Component Analysis (ICA) was computed by using the built-in EEGLAB extended Infomax (runica) decomposition algorithm on the raw data, which were first filtered using a 1 Hz high-pass (Hamming window, filter order 1650) and a 45 Hz low-pass (same parameters as before) windowed FIR filter in order to optimize the ICA decomposition, epoched (− 200 to 600 ms relative to tone presentation), and cleaned by removing the same bad channels and epochs detected at the earlier step. The obtained weights were stored and transferred to the 0.1 Hz high-pass filter datasets. The removal of components containing eye-related and muscle artifacts was done based on visual inspection and paired with the recommendations computed by SASICA, which refer to low auto-correlation of time-course, focal channel topography, focal trial activity, correlation with vertical EOG, and correlation with horizontal EOG^30^. On average, seven principal components (range: 6–8) were removed. The missing channels were interpolated using the built-in EEGLAB spherical interpolation function, and data were baseline corrected using the − 200 to 0 ms interval for the post-stimulus analyses, and the − 850 to − 650 ms interval for the pre-stimulus analysis, respectively. Epochs with amplitudes still exceeding a 200 μV signal change within epoch threshold after the ICA corrections were removed; on average, 9.2 epochs were excluded at this this step (range: 0–66). Finally, condition-specific grand-averages were calculated for the standard and deviant tones, for the post-stimulus analyses. Note that the standard grand-averages excluded each first tone presented after deviants. For the pre-stimulus analyses, the condition-specific grand-averages were calculated for the left and right hand responses (irrespective of whether these subsequently generated standard or deviant tones) and on the data additionally filtered with a 10 Hz low-pass filter (Hamming window, filter order = 414).

### PCA analysis

To determine the ERP components of interest at the post-stimulus intervals, a temporal Principal Component Analysis (PCA) was performed on the grand-average data corresponding to the standard and deviant tones in the *SPEC* and *UNSPEC* conditions, by using the ERP PCA toolkit MATLAB-based toolbox^31^. In accordance with current recommendations^32^, a Geomin rotation (ϵ = 0.05) with a covariance relationship matrix and no weighting was used. Horn’s parallel test further determined the number of components to be retained. Note that each principal component identified by the temporal PCA is characterized by two parameters: first, the time-variant *component loadings* reflect its correlation with the ERP at each point in time, that is, the component time course or activation latency. Second, the time-invariant *component scores* represent the contribution of each component to the observed ERP wave per participant, electrode and condition, that is, the component amplitude.

### Statistical analyses

The component scores of each component of interest identified by the temporal PCA at post-stimulus intervals were separately tested using a 2 × 2 repeated-measures ANOVA with factors Condition (*SPEC* vs. *UNSPEC*) and Stimulus type (Standard vs. Deviant). The LRP pre-stimulus analysis was conducted by using A 2 × 2 × 2 repeated-measures ANOVA with factors Condition (*SPEC* vs. *UNSPEC*), Hand (Left vs. Right), and Laterality (Contralateral vs. Ipsilateral), on the mean amplitudes in the − 350 to − 50 ms window. This more conventional approach regarding a rather large window of interest was preferred due to the exploratory nature of this analysis. The LRP onset latencies were computed on the mean of the contralateral minus ipsilateral difference over hands per condition with an absolute of 0.3 µV criterion^33^, and were compared with frequentist and Bayesian *t*-tests between conditions. The statistical significance was defined at the 0.05 alpha level, and results are reported including the partial eta-square effect sizes (*η*^2^_*p*_). We further investigated the frequentist ANOVA main effects and interaction with corresponding Bayesian *t-*tests. Note that this analysis strategy warrants optimal correspondence between the frequentist and Bayesian comparisons, while additionally allowing evaluating support provided by the data for the null hypothesis as well. However, for conciseness, in the LRP analysis, we only report the main effects and interactions that are significant/provide support for the alternative hypothesis. Significant interactions and/or interactions that provided evidence for the alternative hypothesis were followed-up with (frequentist and corresponding Bayesian) *t*-tests.

For all Bayesian comparisons, the Bayes factor (*BF*_*10*_) was calculated; the null hypothesis corresponded to a standardized effect size δ = 0, and the alternative hypothesis was defined as a Cauchy prior distribution centered around 0 with a scaling factor of *r* = 0.707^34^. In line with the Bayes Factor interpretation^35,36^ and with previous studies reporting Bayes Factors^14,37–39^, data were taken as moderate evidence for the alternative (or null) hypothesis if the *BF*_***10***_ was greater than 3 (or lower than 0.33), while values close to 1 were considered only weakly informative. Values greater than 10 (or smaller than 0.1) were considered strong evidence for the alternative (or null) hypothesis. All statistical analyses have been conducted using the JASP 0.9.1.0 software.

## Results

### Behavioural results

#### Timing errors

These were calculated as error percentages (%ERR) relative to the total number of trials in each condition. In the *SPEC* condition, participants made on average 0.11%ERR (SD = 0.22%, range = 0–0.77%) by pressing the keys much faster than requested, and 0.14%ERR (SD = 0.16%, range = 0–0.49%) by pressing the keys much slower than requested (i.e., less than 500 ms or more than 1500 ms between consecutive key-presses). In the *UNSPEC* condition, participants made on average 0.16%ERR (SD = 0.21%, range = 0–0.66%) by pressing the keys much faster than requested, and 0.3%ERR (SD = 0.52%, range = 0–2.07%) by pressing the keys much slower than requested. Results of a 2 × 2 frequentist ANOVA showed no significant main effects of Condition (*F*(1, 13) = 3.21, *p* = 0.096, *η*^*2*^_*p*_ = 0.198) or Timing (*F*(1, 13) = 3.38, *p* = 0.089, *η*^*2*^_*p*_ = 0.206), nor a significant interaction term (*F*(1, 13) = 0.70, *p* = 0.418, *η*^*2*^_*p*_ = 0.051). To conclude, the overall small percentages of error rates suggest that participants pressed the keys at the suggested pace, while the lack of condition differences suggest that participants’ performance was stable throughout the whole task.

#### Ratio of left/right key-presses

On average, participants pressed the left key on 50.41% (range = 46.76–52.30%) and the right key on 49.59% (range = 47.70–53.24%) of the trials in the *SPEC* condition. Similarly, in the *UNSPEC* condition, the left key was pressed on 50.21% (range = 48.02–53.15%) and the right key on 49.79% (range = 46.85–51.98%) of the trials. Results of a 2 × 2 frequentist ANOVA with factors Condition (*SPEC* vs. *UNSPEC*) and Hand (left vs. right) showed no significant main effects of Hand (*F*(1, 13) = 1.05, *p* = 0.324, *η*^*2*^_*p*_ = 0.075) or interaction of Hand × Condition (*F*(1, 13) = 0.23, *p* = 0.635, *η*^*2*^_*p*_ = 0.018). Thus, the left and right keys were pressed about equally often throughout the whole task.

### ERP PCA results

**Figure 2:**
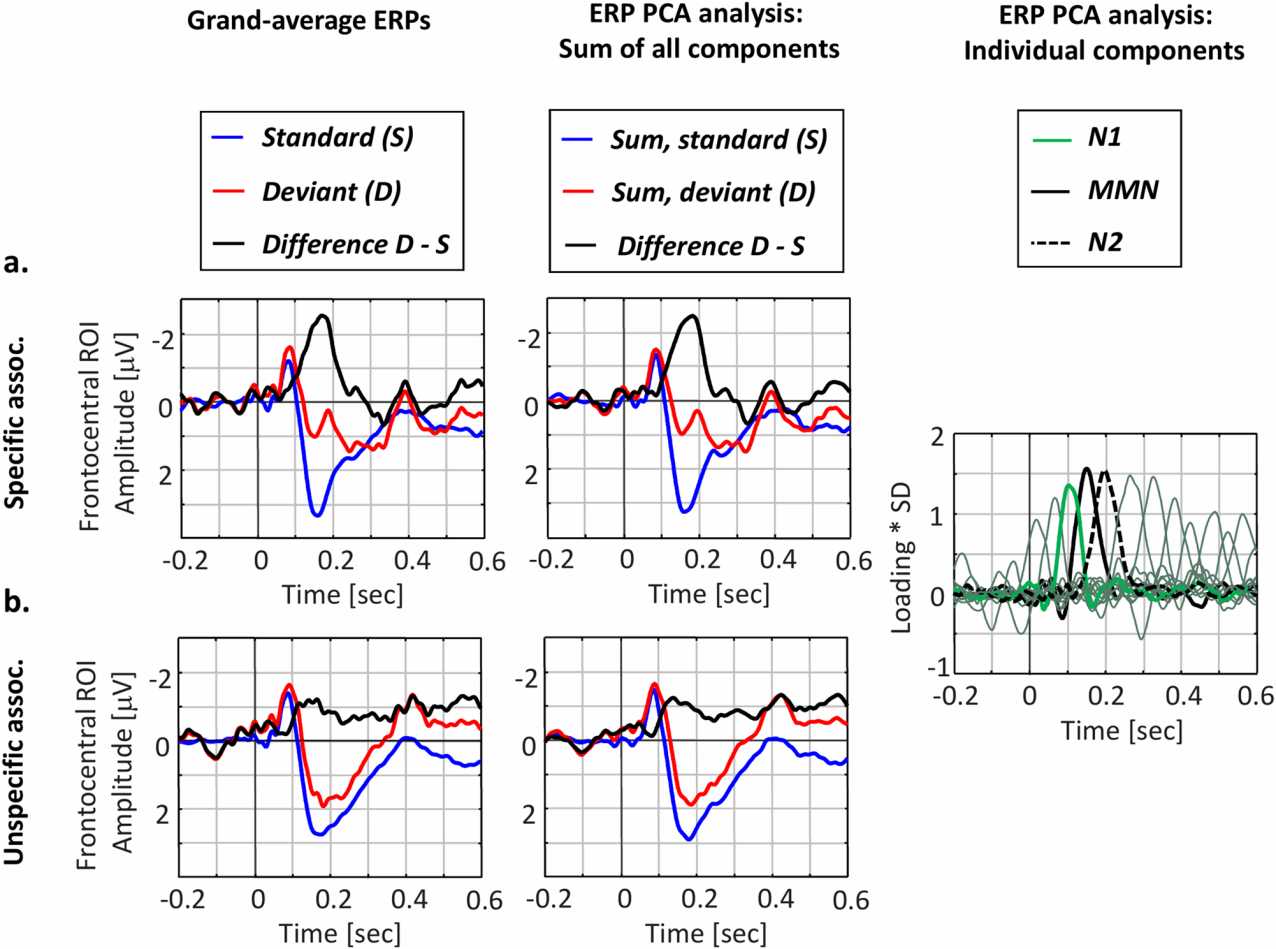
ERP PCA results. Grand-average ERPs (left) display the standard, deviant and difference waves for the conditions with specific (**a**) and unspecific (**b**) associations, for an average of frontocentral Fz, FC1, FC2, Cz, CP1, and CP2 electrodes. Following the PCA analysis, 14 principal components explaining more than 95% of the epoch variability were retained, the sum of these components or the so-called reconstruction waves (middle) being displayed again for the standard, deviant, and difference wave in both conditions, for the same average of electrodes as before. Note that the reconstruction waves correspond well to the grand-average ERPs indicating the PCA solution accurately represents the original data. The 14 retained components are presented individually (right); out of these, three components presumably representing N1, MMN, and N2 responses were further analysed. Figure generated in MATLAB, version R2017a (http://www.mathworks.com/).

The grand-average ERPs along with the PCA results are displayed in Fig. 2, for the specific (Fig. 2a) and unspecific conditions (Fig. 2b). As suggested by Horn’s parallel test, 14 components explaining over 95% of the epoch variability were retained (Fig. 2, last column). The condition-specific grand-average ERPs (Fig. 2, first column) and the reconstructed PCA waves representing the sum of the 14 retained components (Fig. 2, middle column) are displayed together for a region of interest composed of frontocentral electrodes Fz, FC1, FC2, Cz, CP1, and CP2.

Note that the PCA reconstruction waves very well correspond to the grand-average ERPs for the standard, deviant, and difference (i.e., deviant—standard) waves, suggesting that the PCA solution accurately represents the original data. The selection of the components of interest was based on latency and topographical information. Two sub-components of the classical MMN were separated: a peak at 150 ms presumably representing an early MMN response, and a later peak at 196 ms presumably corresponding to the N2 component. A component peaking at 102 ms and corresponding to the N1 response was also identified. Note that the principal components are ordered by explained variance, not by peak latency. That is, the MMN and N2 are represented by components number 4 and 2, respectively, and together explain ~ 22.7% of the epoch variability, while the N1 is represented by component number 7 and explains ~ 2.5% of the epoch variability.

**Figure 3:**
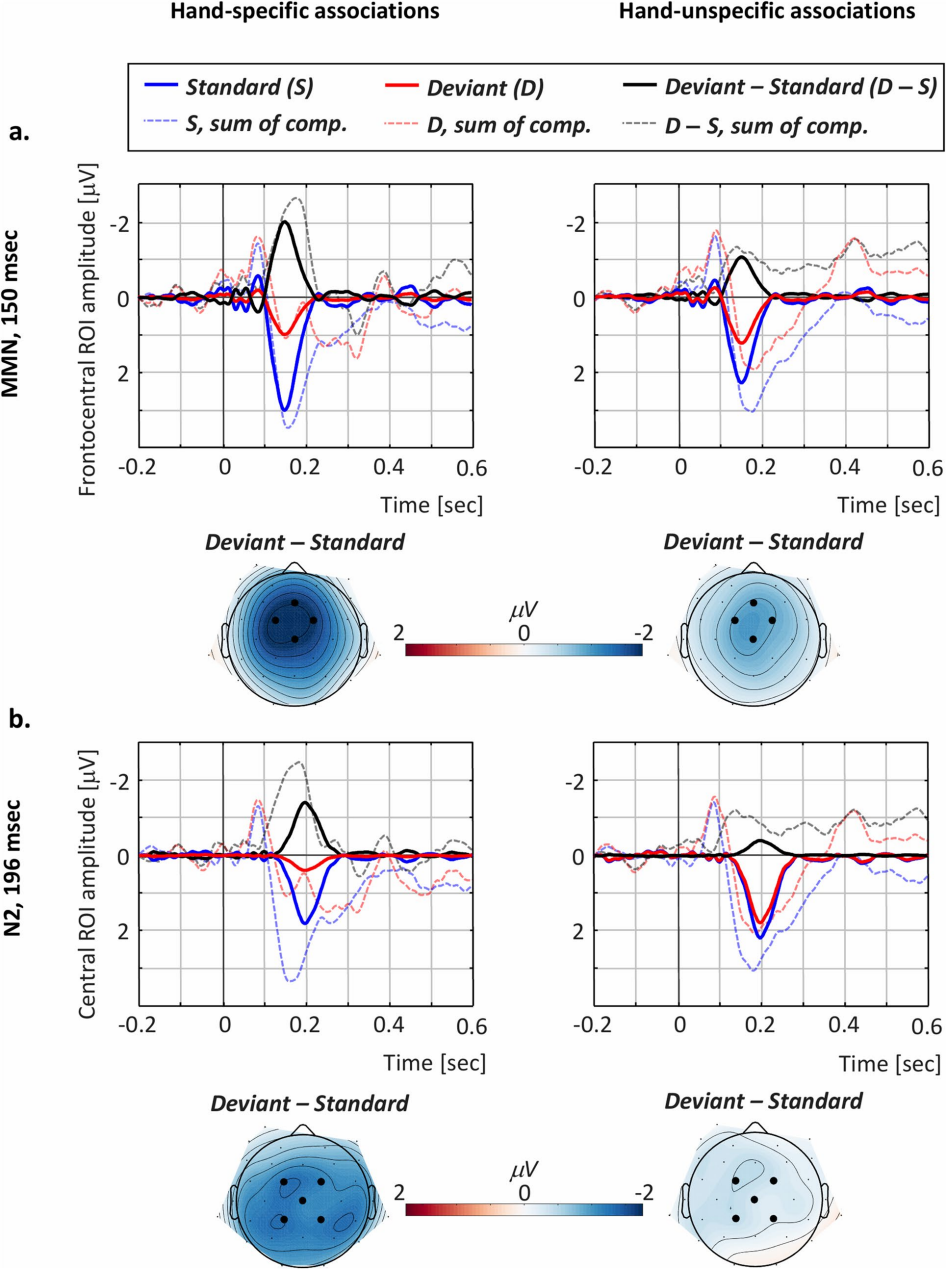
MMN and N2 components. The MMN (**a**) peaks at 150 ms and is largest over a frontocentral region of interest (ROI) composed of the Fz, FC1, FC2, and Cz electrodes, while the N2 (**b**) peaks at 196 ms and is largest over a central ROI composed of the FC1, FC2, Cz, CP1, and CP2 electrodes, the displayed waves representing an average of these. For each component and each condition (specific associations—left; unspecific associations—right), the component-specific activity (solid lines) is displayed along with the reconstruction waves representing the sum of the 14 retained components (dashed, transparent lines) for the standard, deviant, and difference waves. The component- and condition-specific topographical maps illustrate the deviant—standard activation and have been calculated based on spherical spline interpolation. The electrodes marked on the topographical maps represent the component-specific ROIs which were further included in the statistical analyses. Figure generated in MATLAB, version R2017a (http://www.mathworks.com/).

**Figure 4:**
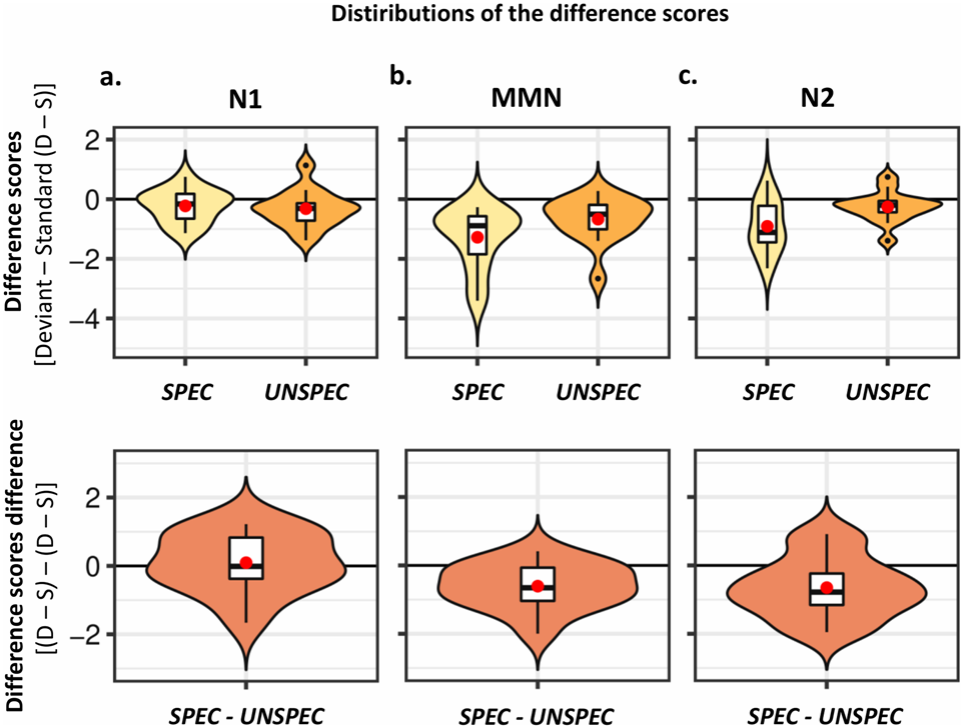
Difference scores. Violin plots display the condition-specific distributions for the difference scores (deviant—standard, top) and for the differences between conditions regarding the observed mismatching effects (deviant—standard differences between conditions, bottom) for the N1 (**a**), MMN (**b**), and N2 (**c**) components. The estimated density distributions are shown along with boxplots indicating the medians, interquartile ranges, and confidence intervals, whereas the means are displayed by the red dots. The black dots represent individual data points falling outside the confidence intervals. Figure generated in R, version 3.5.1 (https://cran.r-project.org/), in combination with the “ggplot2” package (https://ggplot2.tidyverse.org/).

Figure 3 displays the condition-specific waves for the standard and deviant tones, along with the difference waves and their specific topographical maps (deviant—standard activations), for the MMN (Fig. 3a) and N2 (Fig. 3b) components. Additionally, Fig. 4 displays the distributions of the condition-specific difference scores (i.e., deviant—standard) along with a contrast between conditions regarding the observed difference scores (i.e. (*SPEC* deviant—standard)—*UNSPEC* (deviant—standard)) for the N1 (Fig. 4a), MMN (Fig. 4b), and N2 (Fig. 4c) components. The components were analysed at canonical regions of interest (ROIs), in agreement with previous literature regarding the topographies of the N1^7,15^, MMN^6,11,15^, and N2^40^. Specifically, the MMN has a frontocentral distribution largest at electrodes Fz, FC1, FC2, and Cz (see topographical maps in Fig. 3a)—the analyses therefore focused on an average of these, as was also the case for the N1 component (topographical maps not displayed). The N2 component has a central distribution posterior to that of the MMN (see topographical maps in Fig. 3b), the analysis thereby focused on an average of the FC1, FC2, Cz, CP1, and CP2 electrodes. For a summary of the statistical analyses that we report next, please refer to Table 1.

**Table 1:** Results of Statistical Analyses.

Component	Frequentist main effects and interactions and corresponding Bayesian pairwise comparisons				
		*F*	*p*	*η*^*2*^_*p*_	*BF*_*10*_
N1	Condition (*SPEC* vs. *UNSPEC*)	1.49	.244	.103	0.50
	Stimulus type (Std vs. Dev)	**6.41**	**.025**	**.330**	2.70
	Condition × Stimulus type	0.16	.691	.013	0.29
MMN	Condition (*SPEC* vs. *UNSPEC*)	1.20	.292	.085	0.45
	Stimulus type (Std vs. Dev)	**19.79**	** < .001**	**.604**	**56.27**
	Condition × Stimulus type	**11.79**	**.004**	**.476**	**11.10**
N2	Condition (*SPEC* vs. *UNSPEC*)	**11.24**	**.005**	**.464**	**9.75**
	Stimulus type (Std vs. Dev)	**15.23**	**.002**	**.540**	**23.59**
	Condition × Stimulus type	**7.54**	**.017**	**.367**	**3.75**

For each of the three components of interest, a 2 × 2 frequentist ANOVA with factors Condition (*SPEC* vs. *UNSPEC*) and Stimulus type (standard vs. deviant) was computed. Corresponding Bayesian pairwise comparisons tested the magnitude (or the lack) of the evidence regarding the frequentist main effects and interactions (for a detailed description, see “Statistical analyses”). Note that these complementary analyses insure optimal correspondence between the Bayesian and frequentist results, while allowing evaluating support provided by the data for the null hypothesis as well. Significant frequentist effects and *BF*_*10*_ supporting the alternative hypothesis are highlighted in bold.

N1: The frequentist repeated-measures ANOVA lead to a significant main effect of Stimulus type (*F*(1, 13) = 6.41, *p* = 0.025, *η*^*2*^_*p*_ = 0.330) and no significant interaction term (*F*(1, 13) = 0.16, *p* = 0.691, *η*^*2*^_*p*_ = 0.013), nor main effect of Condition (*F*(1, 13) = 1.49, *p* = 0.244, *η*^*2*^_*p*_ =0.103; see Table 1). The Bayesian *t*-test corresponding to the main effect of Stimulus type brought however only weak evidence for the alternative hypothesis (*BF*_*10*_ = 2.7). The Bayesian *t*-tests corresponding to the main effect of Condition (*BF*_*10*_ = 0.5) and interaction term (*BF*_*10*_ = 0.29) brought weak and moderate evidence for the null hypothesis, respectively. Therefore, no reliable N1 effects were observed overall, while the evidence supported the null hypothesis regarding the condition differences (see also Fig. 4a).

MMN: The frequentist repeated-measures ANOVA brought forward a significant main effect of Stimulus type (*F*(1, 13) = 19.79, *p* < 0.001, *η*^*2*^_*p*_ = 0.604) and a significant interaction of Condition × Stimulus type (*F*(1, 13) = 11.79, *p* = 0.004, *η*^*2*^_*p*_ = 0.476), while the main effect of Condition was non-significant (*F*(1, 13) = 1.2, *p* = 0.292, *η*^*2*^_*p*_ = 0.085; see Table 1). The Bayesian *t*-tests corresponding to the main effect of Stimulus type brought strong evidence for the alternative hypothesis (*BF*_*10*_ = 56.27), as did the comparison regarding the interaction term (*BF*_*10*_ = 11.1), while the evidence corresponding to the main effect of Condition weakly supported the null hypothesis (*BF*_*10*_ = 0.45). Following up on the interaction term bringing evidence for the existence of MMN condition differences, the direct comparison of deviant vs. standard tones demonstrated significant elicitation of MMN in both the *SPEC* (*t*(13) = 4.76, *p* < 0.001), as well as in *UNSPEC* conditions (*t*(13) = 3.36, *p* = 0.005). Complementary Bayesian *t*-tests further proved that the alternative hypothesis was strongly supported in the *SPEC* (*BF*_*10*_ = 92.41), and moderately to strongly supported in the *UNSPEC* condition (*BF*_*10*_ = 9.98). To conclude, the data indicated that the MMN component was elicited in both conditions, but stronger in *SPEC* condition (see also Fig. 4b).

N2: The frequentist repeated-measures ANOVA pointed to significant main effect of Condition (*F*(1, 13) = 11.24, *p* = 0.005, *η*^*2*^_*p*_ = 0.464) and Stimulus type (*F*(1, 13) = 15.23, *p* = 0.002, *η*^*2*^_*p*_ = 0.540), as well as to a significant interaction term (*F*(1, 13) = 7.54, *p* = 0.017, *η*^*2*^_*p*_ = 0.367; see Table 1). The Bayesian *t*-tests corresponding to the main effect of Stimulus type brought strong evidence for the alternative hypothesis (*BF*_*10*_ = 23.59), while the evidence corresponding to the main effect of Condition and interaction term moderately supported the alternative hypothesis in both cases (*BF*_*10*_ = 9.75 and *BF*_*10*_ = 3.75, respectively). Following up on the interaction term bringing evidence for the existence of condition differences regarding the N2 effect, the direct comparison of deviant vs. standard tones demonstrated significant elicitation of N2 in the *SPEC* (*t*(13) = 3.91, *p* = 0.002), but not in the *UNSPEC* (*t*(13) = 1.83, *p* = 0.082) condition. This was confirmed by the Bayesian complementary comparisons indicating that the alternative hypothesis was strongly supported in the *SPEC* condition (*BF*_*10*_ = 23.94), while the evidence in the *UNSPEC* condition was uninformative (*BF*_*10*_ = 1.07). Thus, the data pointed towards condition differences regarding the N2 effect, which only seemed to be reliably elicited in the *SPEC* condition (see also Fig. 4c).

### LRP results

**Figure 5:**
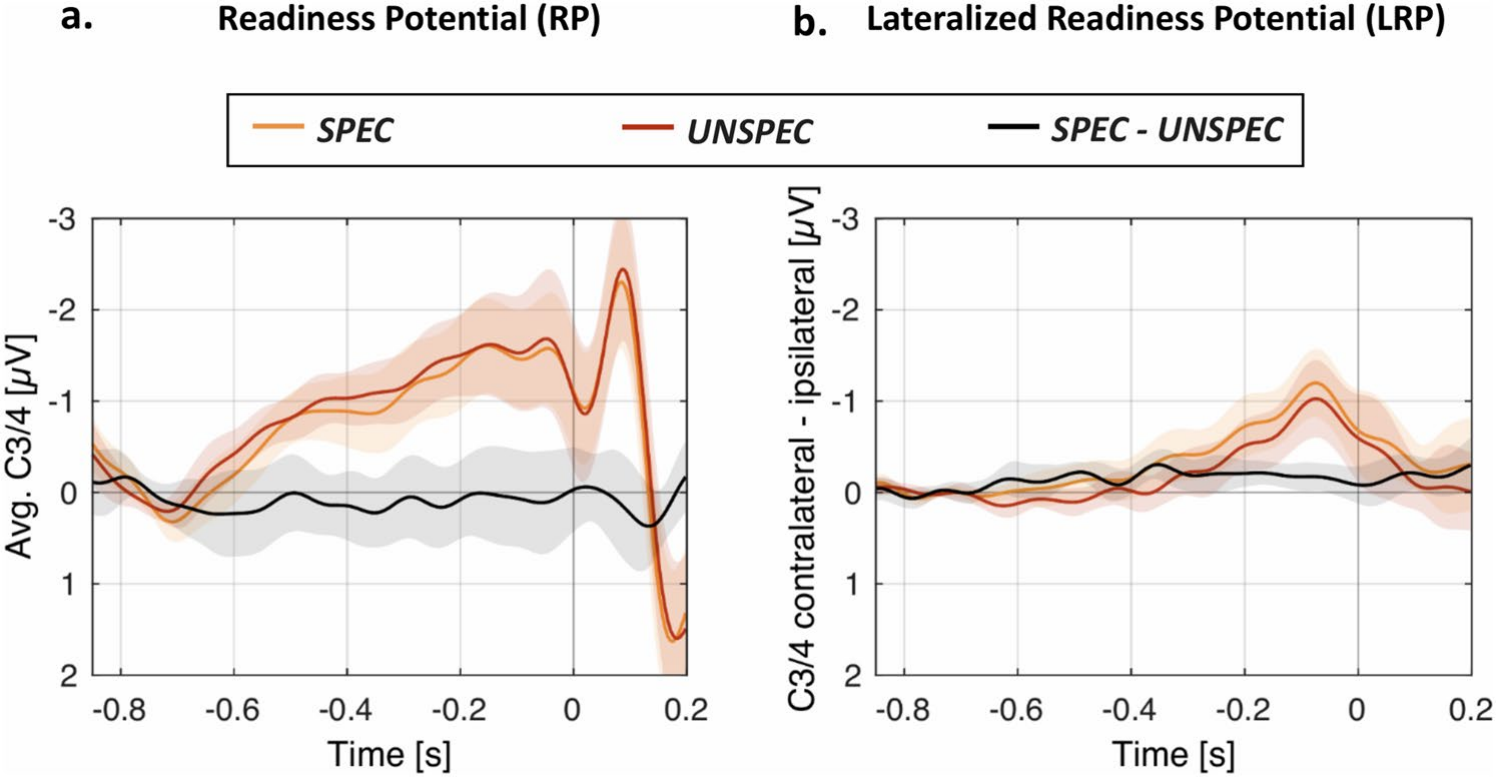
(L)RP results. Grand-average ERPs incl. 95% confidence intervals (shown in transparent colours) display the pre-stimulus activity in the *SPEC* and *UNSPEC* conditions, as well as the *SPEC—UNSPEC* differences, for the Readiness Potential (RP; **a**) referring to an average of C3 and C4 electrodes, and the Lateralized Readiness Potential (LRP; **b**) referring to the contralateral–ipsilateral condition differences. Figure generated in MATLAB, version R2017a (http://www.mathworks.com/).

Figure 5 presents the Readiness Potential (RP; Fig. 5a) along with the LRP (Fig. 5b) component, which was analysed at electrodes C3 and C4, in agreement with current recommendations^41^. The LRP had a more negative mean amplitude (− 0.72 µV vs. − 0.51 µV) and earlier onset latency (− 364 ms vs. − 248 ms) in the SPEC compared to the UNSPEC condition. The frequentist repeated-measures ANOVA on the LRP mean amplitudes lead to a significant main effect of Laterality (*F*(1, 13) = 21.92, *p* < 0.001, *η*^*2*^_*p*_ = 0.628) and a significant interaction of Condition × Laterality (*F*(1, 13) = 14.36, *p* = 0.002, *η*^*2*^_*p*_ = 0.525). Concurring, the Bayesian *t*-tests corresponding to the main effect of Laterality brought strong evidence for the alternative hypothesis (*BF*_*10*_ = 81.01), as did the comparison regarding the of Laterality × Condition interaction (*BF*_*10*_ = 19.65). All other main effects and interactions were non-significant/did not provide support for the alternative hypotheses. Following up on the interaction term bringing evidence for the existence of LRP condition differences, the direct comparison of contralateral vs. ipsilateral responses demonstrated significant elicitation of the LRP component in both the *SPEC* (*t*(13) = 5.20, *p* < 0.001) and *UNSPEC* conditions (*t*(13) = 3.95, *p* = 0.002). Concurring, the complementary Bayesian *t*- tests bring strong support for the alternative hypothesis in both *SPEC* (*BF*_*10*_ = 178.72), and *UNSPEC* (*BF*_*10*_ = 25.32). Additionally, the *t*-test comparing *SPEC* vs. *UNSPEC* LRP onset latencies was statistically significant (*t*(13) = 4.02, p = 0.001)/brought strong support for the alternative hypothesis (*BF*_*1*0_ = 28.62). To conclude, the data indicated that the LRP was elicited in both conditions, but stronger and earlier in *SPEC*.

## Discussion

The predictive coding framework suggests that in order to process the incoming sensory information efficiently, the brain is continuously building up and updating predictive models, where sources for predictions may be drawn from sensory regularities and/or action-effect regularities. Since recent results from the auditory system literature indicate that stochastic regularities (i.e., learning that some tones are more likely to occur than others) seem to be rather difficult to detect if the pitch of two standards enclose the pitch of the deviant tone^15^, the scope of this paper was to test whether associating the two standard tones with intended action-effects would lead to facilitated detection of the enclosed rare tone. Indeed, a larger MMN response was obtained in the *SPEC* condition, by comparison to the *UNSPEC* condition in which the standard tones could not be predicted based on the action choice. Our results thus suggest that intention-based action-effect predictions can enhance the encoding of stochastic regularities, extending previous findings that action intention alone (i.e., in the absence of any auditory regularities) leads to predictions at sensory levels^14^. The data further indicated that higher-order deviance detection processes reflected by the N2 component were reliably engaged when the tones could be predicted based on the action choice (in the *SPEC* condition), but not based on the stochastic regularity alone (in the *UNSPEC* condition). Nevertheless, the necessary and sufficient conditions for the encoding of stochastic regularities is still yet to be determined by future research, as we also observed a weaker MMN when specific predictions based on the action choice were not possible.

Additionally, an exploratory analysis focusing on the LRP component tested whether the specificity of the expected action-effects modulates action preparation. Congruent with our post-stimulus findings, the pre-stimulus LRP was larger when the action choice lead to specific sound predictions, that is, the hand-specific preparatory activity was stronger in the *SPEC* compared to *UNSPEC* condition. According to a previous study^42^, the RP may be increased when key-presses are associated with sounds, by contrast to when they have no sensory consequences. Moreover, action preparation presumably represents a hierarchical process, where the magnitude of the (L)RP depends on whether the precise action parameters such as direction of movement are already known^43^. Our result seems to go one-step further: the LRP magnitude does not only depended on the action parameters or on whether the action has any sensory consequences at all, but it depends on whether it is carried out to determine specific sensory outcomes. The present effect can further be related to the recently-described prediction potential (PP)^44^. The PP is a negative going shift preceding sensory input that can be predicted by contextual information. The RP is also a negative going shift, but preceding an action, rather than a stimulus. Considering that action and perception can be conceptualized in a joint theoretical framework postulating common mental codes for action and perception^45^ and that the intention to press a key makes the action highly predictable, one may regard the present RP as the motor analogue of the sensory PP. Moreover, similarly as the PP reflects perceptual and semantic features of anticipated stimuli before they appear^46,47^, its motor analogue, the RP reflects this “feature” specificity by the fact that it lateralizes (the LRP) with the hand that will execute the action. Finally, while this finding may provide important new directions, further corroboration is needed, given the explanatory nature of this comparison.We next focus on discussing the post-stimulus MMN and N2 findings in more detail.

### Action-effect predictions facilitate the early sensory predictions

Previous findings demonstrated that action-effect predictions can modulate the N1^48^, MMN^14^, N2b^49^, and P3a^14,50^ amplitude, depending on whether the received input can be correctly predicted based on the action choice or not. While some of these studies do not clearly separate the effects of action predictions from those of sensory predictions based on stimulus regularity, one study found that the MMN component was elicited without global regularity, but based on expected action-effects only; that is, the MMN was elicited to violations of the expected action-effects when two tones (inversely associated with left or right key-presses) were overall presented with equal chances^14^. Thus, even though it has been demonstrated that sensory regularity, if available, plays a role in the context of action predictions^37,51–54^, it seems that the action intention alone can be sufficient to drive predictions at sensory levels, when no regularities can otherwise be extracted from the incoming stimulation. In the present study, we found that the magnitude of the MMN was larger in the *SPEC* condition, even though MMN responses were observed in both the *SPEC* and *UNSPEC* conditions. This extends the previous action prediction results and demonstrates that under certain task conditions, predictions based on expected action effects can help boost the early sensory predictions based on encoded environmental regularities. Note the observed MMN effect is clearly post-N1 and presumably represents a “true” prediction marker, rather than neural adaptation-related processes^55^. Specifically, the N1 was detected by the temporal PCA as a separate component for which no reliable modulation was found.

Note however that based on the present data, we cannot conclude whether what we observe as a facilitation effect occurs due to an interaction of (stochastic) regularity-based and action-effect-based predictions, or rather due to independent and thus additive effects of the two predictions types. Recent results^14^ indicated that deterministic regularities and intention might integrate rather than add up, when available simultaneously – this means that once the prediction system gathers enough information (which could be extracted from either regularity or intention), additional input does not change the observed effects. Yet, the relationship between stochastic regularities where the expected input cannot be accurately determined on a trial-by-trial basis and intention might be fundamentally (and functionally) different. While the previously reported integration of action and sensory predictions^14^ may suggest equivalence between the two prediction types^20^, the present results bring forward a new functional role of the action predictions: they may serve to increase the precision gain referring to the reliability of the prediction^56^, in case of (more complex) sensory predictions.

Finally, we would like to point out the possibility that this facilitation effect on the encoding of stochastic regularities might not be specific to action predictions. Previous studies showed that the magnitude of the Incongruency Response (IR), an ERP component indexing visual-auditory predictions^39,57^ may increase in case of concurrent visual-auditory and regularity-based prediction violations^58^. Moreover, previous results indicated that action predictions and sensory visual-auditory predictions lead to comparable effects, indicating that the two information types feed into a common generative model, despite presumably having different brain sources^59^. This would confirm that action predictions are not qualitatively different from sensory predictions^20,21^ and that they are likely to stem from more general cognitive processes, rather than from specific information coming from the motor system^19^. Nevertheless, while most studies focus on identity predictions^8^, additional decisions regarding when and whether to act which in turn modulate the sensory processing of action-effects as well^60,61^, might still make action-related predictions functionally distinctive from other types of sensory predictions.

### Action–effect predictions additionally lead to higher-order deviance detection

In addition to the MMN, we observed a further N2 component, as identified by the principal component analysis (PCA), which is highly reliable at identifying the components of interest, given the complex nature of an ERP wave^62^. The two identified components (i.e., MMN and N2), importantly, were differently modulated in the two conditions: that is, the MMN was elicited in both cases with different magnitudes, while the N2 was reliably elicited in the *SPEC* condition only. Correspondingly, the two are not likely to reflect sub-components of the same classical MMN response that is typically observed as a unitary frontocentral effect. Here, the MMN and N2 effects have distinguishable topographical activations, with the second being more centrally distributed, relative to the first one (see Fig. 3). The later component is thus more likely to reflect later and higher-order deviance detection processes that only seem to be engaged when higher-order predictions based on specific action-effects are available for the system. While the N2 belongs to the same family of processes related to deviance processing and error detection as the MMN, by contrast to the MMN, it presumably represents a marker of higher cognitive control and conscious detection of the deviant sounds^40^. While this explanation seems theoretically satisfactory, a functional distinction between the MMN and N2 components should be further supported by polarity inversion at the mastoid sites for the MMN, but not for the N2 component^63^. This indeed seems to be the case also in the present data (see Supplementary Information).

The lower vs. higher cognitive control of the mismatch detection mechanisms as indexed by the MMN and N2 components respectively is further congruent with the assumed architecture of the predictive brain. Specifically, different generative models are built up, either in a bottom-up fashion, where prediction error is propagated from peripheral to sensory processing areas as in the case of sensory regularity-based predictions, or in a top-down fashion, where predictions regarding the expected input are sent from central to sensory levels. In this context, cognitive control and attentional engagement represent an inherent property of the top-down, but not necessarily of the bottom-up system. Note that we do not suggest that generative models adjusted by bottom-up means cannot draw on attentional resources, as we know this is not true from studies where, for instance, strong regularity-based MMN responses are followed by P3 components^64^, which presumably index the attentional switch to motivationally significant stimuli^65^. What we point out instead is that higher-order processes are only involved if the early detection of regularity violations reach a certain threshold^64^. In the context of our results, this means that in the *UNSPEC* condition, the stochastic regularities were not encoded sufficiently strongly without the additional top-down mechanism based on expected action-effects—therefore, the subsequent N2 component was not reliably elicited.

### Stochastic regularity detection or learned unspecific actions-effects?

Based on previous results^15^, we did not expect to find a MMN effect in the *UNSPEC* condition. Yet, the active nature of the task (i.e., involving increased attentional resources) and the longer SOA (i.e., variable around one second as a function of participants’ self-pacing, by contrast to fixed at 500 ms in the original study^15^) might have afforded the necessary conditions for improved perceptual categorization of the tones, and thus the encoding of the stochastic regularity. Indeed, our ability to distinguish objects between themselves seems to depend on timing. This appears to be true for both low-level perceptual categories as for instance in auditory streaming^66^, as well as for high-level cognitive categories as for instance in dual-task studies showing that participants’ categorizing performance decreases with shorter SOAs^67^.

The results from the additionally recorded passive listening task (see Supplementary Information) are of further relevance here. In short, we found similar MMN responses following stochastic regularities in which participants listened to the sequences generated in the active part and deterministic regularities in which they listened to sequences of alternating standards with random deviants in between. Thus, the argument that stochastic regularity encoding as reflected by the MMN component might be explained due to longer SOA and improved perceptual categorization seems to hold true, also in the passive listening task. Additionally, the passive listening task was always run after the active task, to ensure similar timing across conditions (given by participants’ self-pacing). This means that participants had extensive training regarding the active perceptual discrimination of the tones (in addition to longer timing), which is in turn likely to have contributed to the observed effects, as it has been shown that familiarity modulates the processing of early auditory components^68,69^, also outside the focus of attention^70^. It could finally be argued that the improved attention and perceptual categorization in the present active task might limit the generalization of our results. However, note that in the original study^15^, an additional active condition in which participants had to pay attention to the tones and press a key when detecting the deviant did still not lead to improved detection of the stochastic regularity. Therefore, the explanation is more likely to lie in “true” tone predictability differences (here, based on action), rather than on attentional differences.

Alternatively, recent results coming from omission designs indicate that unspecific action-effects in which the action choice does not accurately determine the tone identity, lead to predictive effects too^37,54^. The flexibility of the predictive system is further demonstrated by its ability to hold concurrent^71^ and even contradictory predictions^58,72^. Thus, in the *UNSPEC* condition, more flexible, binary expectations could have been established for each action-effect, rather than no action-effect expectations at all. Such a binary prediction would in fact represent an action-effect stochastic rule. Yet, regardless of whether the results in the *UNSPEC* condition are considered from a bottom-up (overall tone probability) or top-down (binary action-effects) perspective, the rule remains probabilistic and thus uncertain. By contrast, in the *SPEC* condition, the heard sequences can be regarded as both stochastic from a bottom-up perspective, and more importantly, deterministic from a top-down perspective as on every trial, the identity of the forthcoming tone could be precisely determined based on the action choice. In turn, this leads to an increased prediction confidence and thus larger observed mismatch responses. Note that here, the bottom-up vs. top-down distinction refers to the manner in which the generative models come to be formed and adjusted, rather than how predictions unfold over time once expectations based on the incoming stimulation or established action-effects (or both) exist. Finally, it remains for future studies to determine the precise conditions under which stochastic regularities can be successfully encoded (in active as well as in passive task settings).

## Conclusion

In line with the implication of the predictive coding theory that predictions based on higher-order generative models are fed top-down in the hierarchy to sensory levels, our results demonstrate that intention and learned action-effects enhanced the detection of stochastic regularities in which a rare deviant of medium pitch was enclosed between frequent high and low pitch standards. This was evident at early auditory processing levels as indexed by the MMN component; further congruent with the assumed architecture of the predictive brain, later and higher-order deviance detection processes indexed by the N2 component were reliably engaged only following specific action-effect predictions. These findings therefore add to the literature on the (functional) role of action predictions at sensory levels and point towards new directions on the common mechanisms through which intention and (stochastic) regularities modulate the auditory processing hierarchy. Finally, an exploratory analysis revealed larger and earlier pre-stimulus preparatory activity in the case of specific action-effect predictions, as reflected in the Lateralized Readiness Potential (LRP). Pending further corroboration, the positive relationship between sound predictability and action preparation additionally provides important insights for future action-effect prediction studies.

## Supplementary Information

Supplementary Information.

## Data Availability

The raw EEG datasets generated and analysed during the current study are available in the Zenodo repository, http://doi.org/10.5281/zenodo.4264854, along with the PCA solutions for the active and passive tasks, respectively**.**
