# Supplementary material for: The encoding of stochastic regularities is facilitated by action-effect predictions
Source: Sci Rep. 2021 Mar 24;11:6790. doi: 10.1038/s41598-021-86095-4 (PMC7991673; doi:10.1038/s41598-021-86095-4)
Supplement: Supplementary file 1 — Supplementary Information. [file 41598_2021_86095_MOESM1_ESM.pdf]

Supplementary Information to

The encoding of stochastic regularities is facilitated by action-effect predictions

Betina Korka [1], Erich Schröger [1], Andreas Widmann [1, 2]*

[1]Cognitive and Biological Psychology, Leipzig University, Germany

[2]Leibniz Institute for Neurobiology, Magdeburg, Germany

*Correspondence to:

Betina Korka

Cognitive and Biological Psychology, Institute of Psychology – Wilhelm Wundt, Leipzig University

Neumarkt 9-19

D-04109 Leipzig Germany

Fax: +49 341 97-39271

Email: betina-christiana.korka@uni-leipzig.de

ORCID iD: https://orcid.org/0000-0002-2702-9108

1. The passive listening task: Method

This part of the experiment was always run after the active task. Participants watched "Home", a documentary that was silently played, while listening to sequences of tones in two separate conditions. In the condition with *deterministic regularities* (hereafter, *DREG*) the 900-Hz and 1100-Hz standard tones were presented in alternation while rarely, one of the two standard tones was replaced by the 1000-Hz deviant. The ratio of standard-to-deviant tones was 90% – 10%, with 45% for each of the standard tones. In the condition with *stochastic regularities* (hereafter, *SREG*), the order of the two standard and deviant tones was random, while the overall high (90%) or low (10%) probabilities of the standard and deviant tones were the same as in *DREG*. The *SREG* sequences represented a passive replay of the tones

produced in the active task; essentially, every participant listened to the tones that they previously generated in the *SPEC* condition. The timing in the *SPEC* condition further determined the stimulus-onset-asynchrony (SOA) in both the *SREG* and *DREG* conditions. That is, the tones were presented at the same pace of about one second that every participant had previously generated. Note that trials containing timing errors in the active task were not included in calculating the SOAs, thus a tone was always presented after a minimum of 500 ms and a maximum of 1500 ms following the preceding tone.

Similarly to the active task, the passive listening task consisted of 20 experimental blocks, 10 for every condition, the duration of one block was about 1.5 min, and participants could take self-paced breaks in between. Experimental blocks again consisted of 90 standard tones and 10 deviant tones, 900 and 100 trials being thus collected in every condition for the standard and deviant tones, respectively. The condition order was implemented as follows. Out of the seven participants who started with *SPEC* in the active task, four started with *DREG*, and three started with *SREG* the in the passive task. Regarding the seven participants who started with *UNSPEC* in the active task, two started with *SREG*, and five with *DREG* in the passive task. In total, nine participants started with *DREG* and five with *SREG*, in the passive task. We have initially planned to collect a larger dataset involving full counterbalancing of the four conditions' order across both tasks; however, this was not possible due to the start of the COVID-19 pandemic.

Regarding the EEG data recording and preprocessing, the same steps as for the active task were applied (see main manuscript body). In order to determine the ERP components of interest, a temporal Principal Component Analysis (PCA) was performed on the grand-average data corresponding to the standard and deviant tones in the *DREG* and *SREG* conditions, with the same parameters as in the active task (see main manuscript body for details).

2. The passive listening task: Results and Conclusion

The grand-average ERPs along with the PCA results are displayed in Supplementary Figure S1, for the deterministic (Supplementary Figure S1a) and stochastic (Supplementary Figure S1b) regularities. Similarly to the active task, the condition-specific grand-averages and the reconstructed PCA waves representing the sum of 20 retained components are displayed together for a region of interest composed of frontocentral electrodes Fz, FC1, FC2, Cz, CP1, and CP2. Again, the PCA separated the MMN and N2 components peaking at 156 and 192 ms, respectively; the N1 was represented by another component peaking at 92 ms. The MMN and N2 components explain together ~49,3%, while the N1 explains ~11,6 of the

whole epoch variance. As before, the statistical analysis focused on the component scores of the components of interest (i.e., N1, MMN, and N2), where the same regions of interest as for the active part analyses were used (see main manuscript body). Supplementary Figure S2 displays the condition-specific waves for the standard and deviant tones, along with the difference waves and their specific topographical maps (deviant – standard activations), for the MMN (Supplementary Figure S2a) and N2 (Supplementary Figure S2b) components. Supplementary Table S1 presents a summary of the statistical results, which we report next.

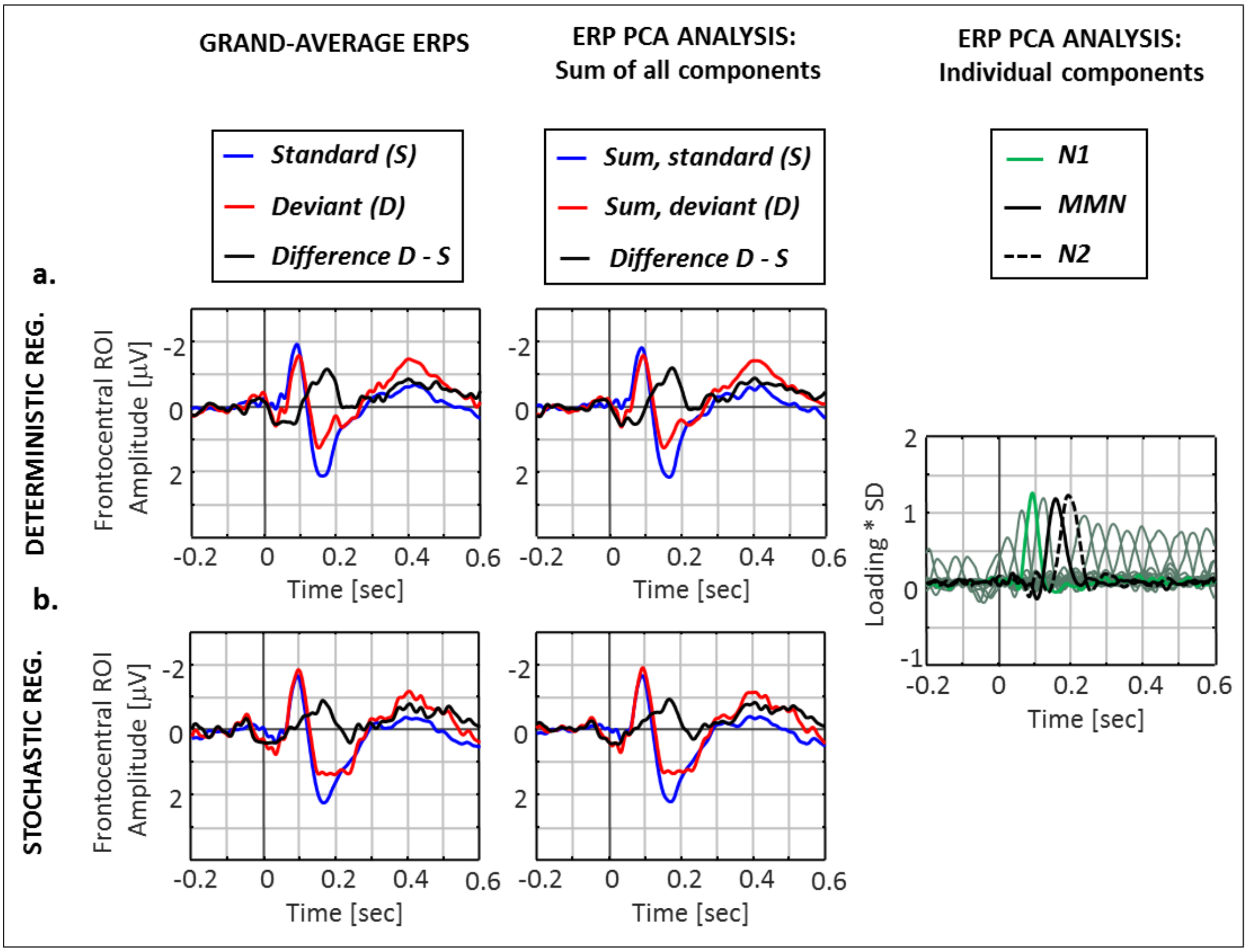

Supplementary Figure S1. ERP PCA results. Grand-average ERPs (left) display the standard, deviant and difference waves for the deterministic (**a**) and stochastic (**b**) regularities, for an average of frontocentral Fz, FC1, FC2, Cz, CP1, and CP2 electrodes. Following the PCA analysis, 20 principal components explaining more than 95% of the epoch variability were retained, the sum of these components or the so-called reconstruction waves (middle) being displayed again for the standard, deviant, and difference wave in both conditions, for the same average of electrodes as before. Note that the reconstruction waves correspond well to the grand-average ERPs indicating the PCA solution accurately represents the original data. The 20 retained components are presented individually (right); out of these, three components presumably representing N1, MMN, and N2 responses were further analysed. Figure generated in MATLAB, version R2017a (http://www.mathworks.com/).

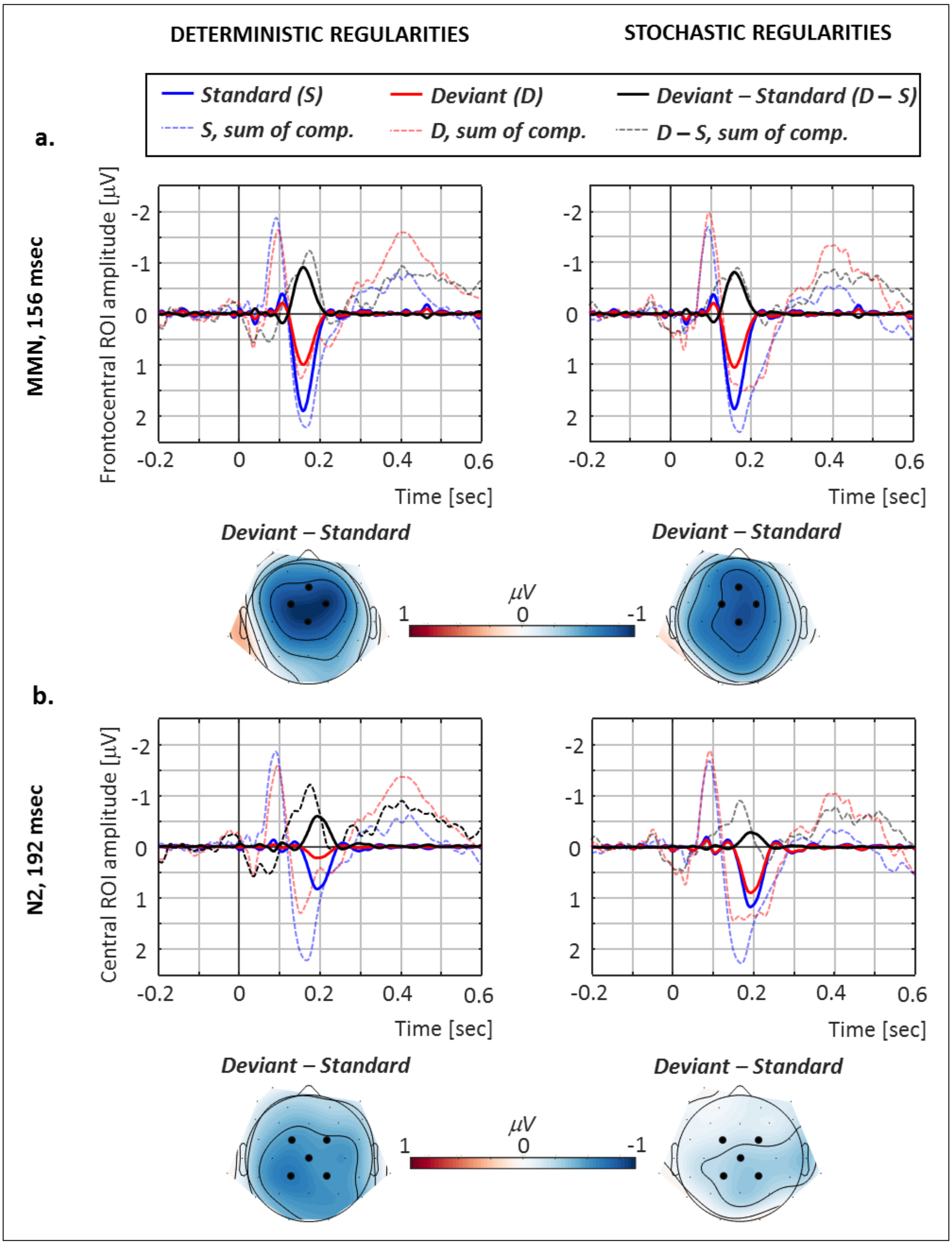

Supplementary Figure S2. MMN and N2 components. The MMN (**a**) peaks at 156 ms and is largest over a frontocentral region of interest (ROI) composed of the Fz, FC1, FC2, and Cz electrodes, while the N2 (**b**) peaks at 192 ms and is largest over a central ROI composed of the FC1, FC2, Cz, CP1, and CP2 electrodes, the displayed waves representing an average of these. For each component and each condition (deterministic regularities – left; stochastic regularities – right), the component-specific activity (solid lines) is displayed along with the reconstruction waves representing the sum of the 20 retained components (dashed, transparent lines) for the standard, deviant, and difference waves. The component- and condition-specific topographical maps illustrate the deviant – standard activation and have been calculated based on spherical spline interpolation. The electrodes marked on the topographical maps represent the component-specific ROIs which were further included in the statistical analyses. Figure generated in MATLAB, version R2017a (http://www.mathworks.com/).

N1: No significant main effects or interactions were brought forward by the frequentist repeated-measures ANOVA (see Supplementary Table 1). Similarly, all Bayesian t-tests corresponding to the main effects and interaction brought moderate to weak support for the null hypothesis (see Supplementary Table 1). Therefore, no N1 effects (nor relevant condition differences) were observed in these data.

MMN: The frequentist repeated-measures ANOVA lead to a significant main effect of Stimulus type ($F(1, 13) = 15.7$, $p = 0.002$, $\eta^2_p = 0.547$), but no significant main effect of Condition ($F(1, 13) < 0.001$, $p = 0.990$, $\eta^2_p = 0.000$) nor interaction term ($F(1, 13) = 0.19$, $p = 0.667$, $\eta^2_p = 0.015$; see Supplementary Table 1). The Bayesian *t*-tests corresponding to the main effect of Stimulus type brought strong evidence for the alternative hypothesis ($BF_{10} = 25.96$), while the evidence corresponding to the main effect of Condition and interaction term moderately supported the null hypothesis in both cases ($BF_{10} = 0.27$ and $BF_{10} = 0.29$, respectively). Thus, the data indicates that the MMN was elicited in both conditions without difference.

N2: The frequentist repeated-measures ANOVA yielded a significant main effect of Condition ($F(1, 13) = 12.05$, $p = 0.004$, $\eta^2_p = 0.481$) and only non-significant trends regarding the main effect of Stimulus type ($F(1, 13) = 4.57$, $p = 0.052$, $\eta^2_p = 0.260$) and the interaction term ($F(1, 13) = 3.19$, $p = 0.097$, $\eta^2_p = 0.197$). The Bayesian *t*-tests corresponding to the main effect of Condition confirmed strong support for the alternative hypothesis ($BF_{10} = 11.81$), while the evidence corresponding to the main effect of Stimulus type was only weakly informative ($BF_{10} = 1.52$) and the interaction term was uninformative ($BF_{10} = 0.95$; see Supplementary Table 1). Thus, although the N2 effect seems to be larger in the *DREG* condition (see Supplementary Figure 2b), the statistical evidence does not support any reliable N2 effects.

To sum up, we found similar MMN responses following the violation of stochastic and deterministic regularities. Note that these effects were clearly post-N1, which was detected as a separate component for which no reliable modulation was found; thus, the observed MMN responses do not represent neural adaptation, but presumably “true” prediction-related processes[1]. Additionally, a non-significant/weak difference between the two conditions at the level of the N2 component indicates that deterministic regularities might still be somewhat easier to recognize. This would be congruent with earlier results from the original study[2] indicating a difference between the two regularity types, especially if we consider that the MMN window in the original study was close to the N2 time range (where the ERP

components were not derived via temporal PCA). The non-significant/weak (by contrast to stronger) N2 difference could be further explained by the fact that in this study, in the deterministic condition, a small (unexplained) positivity for the deviants just preceding the MMN and N2 (see Supplementary Figure S2) may have artificially reduced the deterministic mismatch effects and consequently the difference between the two regularity types. In conclusion, it remains for future studies to clarify the differences between stochastic and deterministic regularity encoding.

Supplementary Table S1. Results of Statistical Analyses: Passive Task

Component	Frequentist main effects and interactions and corresponding Bayesian pairwise comparisons				
		F	*p*	η^2_p	BF_{10}
N1	Condition (*SREG* vs. *DREG*)	0.14	.707	.011	0.28
	Stimulus type (Std vs. Dev)	0.07	.792	.006	0.27
	Condition × Stimulus type	2.05	.175	.136	0.62
MMN	Condition (*SREG* vs. *DREG*)	<.001	.990	.000	0.27
	Stimulus type (Std vs. Dev)	**15.70**	**.002**	**.547**	**25.96**
	Condition × Stimulus type	0.19	.667	.015	0.29
N2	Condition (*SREG* vs. *DREG*)	**12.05**	**.004**	**.481**	**11.81**
	Stimulus type (Std vs. Dev)	4.57	.052	.260	1.52
	Condition × Stimulus type	3.19	.097	.197	0.95

For each of the three components of interest, a 2 x 2 frequentist ANOVA with factors Condition (*DREG* vs. *SREG*) and Stimulus type (Standard vs. Deviant) was computed. Corresponding Bayesian pairwise comparisons tested the magnitude (or the lack) of the evidence regarding the frequentist main effects and interactions (similarly to the main manuscript analyses). Note that these complementary analyses insure optimal correspondence between the Bayesian and frequentist results, while allowing evaluating support provided by the data for the null hypothesis as well. Significant frequentist effects and BF_{10} supporting H1 are highlighted in **bold**.

3. Analyses of mastoid data

In order to distinguish the MMN from the N2 component, we analysed the mastoid data to check whether the MMN, but not the N2, inverts polarity at these sites[3]. Correspondingly, we analysed the average activity of the left and right mastoid electrodes in each of the *SPEC* and *UNSPEC* conditions, for each of the MMN and N2 components. Frequentist repeated-

measures ANOVAs and corresponding Bayesian *t*-tests were performed as described in the *Statistical Analyses* section of the main manuscript.

MMN: The frequentist repeated-measures ANOVA lead to a significant main effect of Stimulus type ($F(1, 13) = 28.26$, $p <0.001$, $\eta^2_p = 0.685$; the Bayesian corresponding comparison also confirmed strong support for the alternative hypothesis, $BF_{10} = 214.59$), indicating polarity inversion at the mastoid sites (standard > deviant across conditions). The main effects of Condition as well as the interaction of Condition × Stimulus type lead to non-significant results ($F(1, 13) = 0.19$, $p = 0.736$, $\eta^2_p = 0.009$ and $F(1, 13) = 0.71$, $p = 0.414$, $\eta^2_p = 0.052$, respectively; the corresponding Bayesian comparisons indicated moderate and moderate-to-weak support for the null hypotheses, $BF_{10} = 0.28$ and $BF_{10} = 0.36$, respectively).

N2: The frequentist repeated-measures ANOVA did not lead to a significant main effect of Stimulus type ($F(1, 13) = 1.68$, $p = 0.217$, $\eta^2_p = 0.115$; the corresponding Bayesian comparison indicated weak support for the null hypotheses, $BF_{10} = 0.54$), indicating no polarity inversion at the mastoid sites (standard ≯ deviant across conditions). The main effects of Condition as well as the interaction of Condition × Stimulus type were also non-significant ($F(1, 13) = 0.89$, $p = 0.361$, $\eta^2_p = 0.064$ and $F(1, 13) = 0.32$, $p = 0.576$, $\eta^2_p = 0.025$, respectively; the corresponding Bayesian comparisons indicated moderate-to-weak support for the null hypotheses, $BF_{10} = 0.39$ and $BF_{10} = 0.31$, respectively).

To conclude, a polarity inversion at the mastoid sites has been observed for MMN component, but not for N2 component.

References

1. Jacobsen, T., & Schröger, E. Is there pre-attentive memory-based comparison of pitch? *Psychophysiology* **38,** 723–727 (2001).

2. Schröger, E., & Roeber, U. Encoding of deterministic and stochastic auditory rules in the human brain: The mismatch negativity mechanism does not reflect basic probability. *Hear. Res.*, 107907 (2020).

3. Ritter, W. *et al*. Event-related potentials to repetition and change of auditory stimuli. *Electroencephalogr. Clin. Neurophysiol.* **83,** 306–321 (1992).
